# The role of technology and screen media use in treatment outcomes of children participating in a digital mental health intervention: a retrospective analysis of Bend Health

**DOI:** 10.3389/fdgth.2025.1556468

**Published:** 2025-05-02

**Authors:** Kelsey McAlister, Darian Lawrence-Sidebottom, Donna McCutchen, Monika Roots, Jennifer Huberty

**Affiliations:** ^1^ Fit Minded, Inc., Phoenix, AZ, United States; ^2^ Bend Health Inc., Madison, WI, United States

**Keywords:** anxiety, depression, attention-deficit hyperactivity disorder, therapy, behavioral health coaching, cognitive behavioral therapy

## Abstract

**Introduction:**

Digital mental health interventions (DMHIs) show promise in improving children's mental health, but there is limited understanding of how technology and screen media influence treatment outcomes. The purpose of this study was to leverage retrospective data to explore the relationships of technology and screen media use with mental health symptoms among children participating in a pediatric DMHI.

**Methods:**

Children ages 6–12 years who participated in a DMHI, Bend Health Inc, in the United States were included. Caregivers reported their child's screen media use and mental health symptoms every 30 days. Associations of screen media use with mental health symptoms were examined at baseline and throughout DMHI participation.

**Results:**

Nearly all children (98.0%) used screen media, with 58.3% exhibiting problematic use and 23.2% showing elevated use at baseline. Elevated screen media use was associated with more severe depressive (z = 2.19, *P* = .022) and anxiety symptoms (z = 2.36, *P* = .019) at baseline, though associations differed by type. Video streaming, internet use, and gaming were linked to inattention, hyperactivity, and oppositional behavior (P's < 0.05). While screen media use decreased for most children during care (93.1%), those with elevated use showed marginally greater improvements in anxiety (z = −1.87, *P* = .062) and inattention symptoms (z = −1.90, *P* = .058).

**Discussion:**

Findings suggest a nuanced interaction between technology use and DMHIs. Future research should explore the specific contexts of screen media use to optimize DMHI effectiveness and address the potential adverse effects of certain screen media activities.

## 1 Introduction

Technology and screen media use, often referred to as ’screen time’ and commonly measured as the total amount of time spent engaging with various devices, among children has surged in recent years, reflecting the rapid advancement and integration of digital devices into everyday life. More than half (53%) of children in the United States own a smartphone by age 11, and 75% under the age of 8 have access to a “smart” mobile device (e.g., smartphone, tablet) (1, 2). Children ages 8–12 spend just under five hours per day on screen media (e.g., social media, watching television), and the amount of time spent watching videos (other than television) has more than doubled since 2015 for this age group (1). Video game use is also high, as more than 90% of children over the age of 2 play video games, three quarters of American households own a video game console, and children ages 8–12 play an average of 1.5–2 hours of video games per day (3, 4). This widespread adoption of technology has fundamentally changed how children interact with their environment and peers and how they access information, highlighting the importance of understanding its impact on their well-being.

Children's mental health has been declining alongside the growth in technology use, with increasing rates of psychological issues including anxiety and depression. In 2020, 5.6 million American youth ages 6–17 years were diagnosed with anxiety and 2.4 million with depression (5), with rates of anxiety and depression increasing in more recent years likely due to the COVID-19 pandemic (6). In addition, 11% of youth in the United States are affected by Attention-deficit/hyperactivity disorder (ADHD), marked by persistent symptoms of inattention, hyperactivity, or both (7). Research indicates that ADHD often co-occurs with oppositional defiance disorder (ODD), as they share common etiology characterized by disrupted executive function and inhibitory control (8). Despite these growing mental health issues among children, one in 5 children do not receive adequate treatment for their mental health (5), and national data suggests that younger children (5–11 years) are less likely to receive mental health treatment compared to older children (12–17 years) (9). Given the state of children's mental health, it is essential to identify and understand the factors influencing their well-being to develop effective interventions.

Concerns have emerged about the potential for technology to have a negative impact on children's mental health. A recent meta-analysis among 40 studies found that, in children under the age of 12, there were weak but significant associations between higher screen media use and internalizing behaviors (e.g., anxiety, depression), emphasizing the need for more longitudinal evidence (10). Some longitudinal studies have further highlighted that elevated screen media use among children aged 2–6 and 9–10 years is linked to poorer emotional well-being and increased internalizing symptoms two years later (11, 12). A randomized controlled trial demonstrated that a reduction in time using screen media for leisure over two weeks led to notable improvements in internalizing symptoms among children aged 6–10 years (13). Additionally, recent evidence has shown that higher screen media is linked to worse ADHD symptoms and higher occurrence of ODD (14, 15).

While there is evidence of the potential harms of screen media use, the relationship between screen media use and mental health remains complex and highly debated. For example, technology facilitates greater access to educational tools and enhanced social connections (16–18). One factor contributing to inconsistencies in findings is the varying definitions of excessive or problematic screen media use across studies. Some frameworks conceptualize problematic use through behavioral addiction models, emphasizing symptoms such as loss of control, withdrawal-like effects, and persistent use despite negative consequences (19). Others take a broader perspective, defining problematic use as excessive screen engagement that displaces essential activities such as sleep, physical activity, or offline social interactions (20, 21). These definitional differences make it challenging to compare studies and may explain why findings on screen media use and mental health appear mixed. Some studies suggest associations between increased screen media use and increased symptoms of anxiety, depression, ADHD, and oppositional behaviors (14, 15, 22–27). Conversely, other research indicates that these associations are small, inconsistent, or influenced by additional factors such as family environment, content type, and individual differences (28–30).

Despite these broad insights, a significant gap remains in the literature. Many studies do not differentiate between various types of screen media children use, often focusing either on overall screen time or on a single type, such as social media, video games, or internet use. Screen use also varies in purpose and engagement level, with passive activities (e.g., watching videos) potentially having different effects on mental health compared to more interactive or social forms of use (e.g., video gaming, educational apps) (31, 32). The few studies addressing these nuances suggest that mental health outcomes may vary depending on the type of screen media use (31–34), highlighting this area as an important gap in existing literature.

Additionally, little is known about how digital mental health interventions (DMHIs)—i.e., mental health support (e.g., therapy) delivered via a digital platform—interact with children's broader technology habits. DMHIs have emerged as a popular and accessible option for the treatment of children's mental health. Although there are digital interventions that directly target problematic screen media use (35–37), to our knowledge, none have explored associations between changes in screen media use in the context of a DMHI targeting mental health challenges (e.g., depression and anxiety). Understanding the downstream benefits of DMHIs on various aspects of mental health and behavior, including changes in screen media use and how these changes influence treatment outcomes, is important for optimizing the effectiveness and personalization of DMHIs to improve mental health outcomes of children. It can also inform the development of best practices for incorporating screen media use management into DMHIs.

Taken together, there is a need to disentangle the associations of screen media use with mental health outcomes in children. By doing so, we can better identify the factors that influence treatment efficacy and develop more targeted and effective interventions that address children's mental health. Therefore, the purpose of this study was to understand technology and screen media use, measured as the total time spent using various screen-based devices, in children seeking treatment from a DMHI, identify associations between these behaviors and mental health symptom severity at baseline, and explore how elevated use of screen media is associated with treatment outcomes longitudinally over the course of the DMHI.

## 2 Materials and methods

### 2.1 Study design and participants

This study was a retrospective analysis of technology and screen media use, as well as mental health outcomes, of children receiving mental health care from a collaborative care DMHI (Bend Health Inc.). Children (ages 6–12 at the beginning of care) were eligible for the study if their caregivers completed the technology and screen media survey before beginning care with Bend Health, and if they participated in care with Bend Health between December 19th, 2023, and December 19th, 2024 (*n* = 2,835). During enrollment, caregivers agreed to Bend Health's Terms and Conditions, in which they agreed to their data (as well as their child's data) being used for general purposes, including research and analysis. Data were de-identified prior to analysis. Study procedures were approved by the Biomedical Research Alliance of New York (Study 23-12-034-1374). This retrospective study was determined “exempt.” Therefore, while participants agreed to their data being used for general purposes, they did not explicitly agree to their inclusion in this study (given its retrospective nature).

### 2.2 Treatment

Bend Health is a collaborative care DMHI that delivers care to children and their families via synchronous (video-based) sessions with mental health providers, including behavioral care managers (BCMs), behavioral health coaches (herein referred to as “coaches”), and licensed therapists, as described previously (38, 39). Children may be referred to Bend Health by their primary care provider, or they may access services through other routes (e.g., insurance). Caregivers of children enroll using Bend Health's secure web-based platform before beginning care, where they provide demographic information and complete mental health assessments, as further described in the “Measures” section.

After enrollment procedures are completed, each child (and their caregiver/s) is assigned a BCM, who continually monitors the child's care with other Bend health providers—including a coach, therapist, or both—as based on their treatment needs and services desired, among other factors (e.g., insurance coverage). The BCM meets with the child and their caregiver/s in an intake session, where they determine the child's care team, treatment goals, and services. Coaches lead a child's care with Bend Health and are certified behavioral coaches or masters-level mental health professionals trained in various standard behavioral techniques, including cognitive behavioral therapy (CBT), dialectical behavioral therapy (DBT), parent management, and mindfulness-based stress reduction. Licensed therapists are equipped with additional skills and clinical experience to manage complex cases and provide a clinical framework to a member's care and are assigned on an as-needed basis or where coverage applies. Members with psychiatric referral or those in need of medication management may also see a psychiatric provider.

In synchronous video-based sessions the practitioner delivers evidence-based tools to the child and their caregiver(s). The content of each session is determined based on structured care programs (assigned given a treatment target), which are module-based programs designed to target specific symptom domains (e.g., depressive symptoms) in an age-appropriate manner. The child and caregiver can also access the content of each care program in a learning resource center to facilitate the development of skills between sessions, and caregivers have access to secure messaging with their child's care providers. Given Bend's focus on whole-family care, caregivers are given specific parenting and support tools to best address their child's needs. Caregivers of children are also required to attend synchronous sessions with their child to ensure their safety.

### 2.3 Study measures

At enrollment, caregivers provide their child's demographic information, unless already provided at referral, including date of birth, sex at birth (“Male”, “Female”, or “Other”), and race/ethnicity (see Supplementary Material 1.1 for details). Then, they complete mental health assessments. First, they respond to mental health screener questions to identify mental health and behavioral concerns, including technology and screen media use, and anxiety and depressive symptom severity. If responses to the screener questions flag a potential mental health or behavioral problem, the caregiver completes a comprehensive survey to measure symptoms. Caregivers whose child continues participating in care with Bend Health complete follow-up mental health assessments every month (within the web-based platform) to regularly monitor outcomes.

#### 2.3.1 Technology and screen media survey

The technology and screen media screener and survey are investigator-developed based on existing surveys (40, 41). The screeners and survey are described in further detail in the Supplementary Material 1.1.1. In brief, technology is defined to the caregiver as including texting, social media, other smart-phone apps, the internet, watching or streaming movies/videos/TV, and gaming, per research by Nagata and colleagues (40). The first screener question inquires about problematic use of technology (Yes/No), with the question: “Do you think your child is dependent (e.g., disturbs their daily life, they can't wait to use it again, and/or overuse) on technology.” The second screener question asks the caregiver to estimate approximate hours per day of technology use (whole-number numerical response 0–24). If caregivers respond “Yes” to problematic use or greater than 0 hours of use per day (screen-in), they complete six more questions about daily screen media use (within the past month) of the following screen media types: texting, social media, other smart-phone apps, the internet, watching or streaming movies/videos/TV, and gaming. The response options are: “My child does not use this type of technology”, “1 hour or less a day”, “More than 1 hour but less than 4 h”, “More than 4 hours but less than 7 h”, “More than 7 h”. Mental health symptom severity.

#### 2.3.2 Mental health symptoms

Depression, anxiety and inattention screener questions are derived from the Diagnostic and Statistical Manual of Mental Disorders, fifth edition, (DSM-V) text revision Cross-Cutting Symptom Measure for children aged 6–17 years (42). The depression screener consists of two questions, and the anxiety screener is three questions. The single inattention screener question screens for both inattention and hyperactivity symptoms, given that these symptoms are often co-occurring (e.g., in individuals with ADHD). Opposition (problematic behaviors) is screened using a question about the child's problematic behaviors in relation to others. Responses to all screener questions are made on a five-item Likert scale with responses ranging from “Not at all” (score = 0) to “Nearly every day” (score = 4). If the response to the depression or anxiety screener questions is two (“Several days”) or greater, the caregiver completes the corresponding validated PROMIS Emotional Distress assessment (43, 44). If the response to either inattention/hyperactivity or opposition screener question is one (“Rare, less than a day or two”) or greater, the caregiver completes the whole SNAP-IV assessment (45). Screeners and assessments are described in further detail in the Supplementary Material 1.1.2.

### 2.4 Statistical analysis

Participant inclusion and respective sample sizes for all analyses are included in Supplementary Material 1.2.1. For all analyses of the technology and screen media survey, use of a type of screen media was indicated by any response other than “my child does not use this type of technology” or screening-out. “Elevated” use was indicated by a response indicating more than four hours of use per day, to reflect moderate to high use of technology and screen media. In previous research, youth who engaged in more than 4 hours of daily screen time were twice as likely to have been diagnosed with depression or anxiety compared to those with less screen time (41). inclusion and respective sample sizes for all analyses are included in Supplementary Material 1.2.1. For all analyses of the technology and screen media survey, use of a type of screen media was indicated by any response other than “my child does not use this type of technology” or screening-out. “Elevated” use was indicated by a response indicating more than four hours of use per day, to reflect moderate to high use of technology and screen media (41). Mental health symptom severity was determined for all symptoms, per standard procedures (43–45), with symptom severity categories ordered as follows: none to slight (screened-out or low assessment score), mild, moderate, and severe (see Supplementary Material 1.2.2 for details).

Descriptive statistics were used to describe participant age, sex, and race and ethnicity. Technology and screen media use at enrollment (baseline) was described as follows: hours of technology use per day [median (interquartile range; IQR)], rates of problematic technology use, and rates of any use and elevated use for each type of screen media. Cumulative link models (CLM) were used to determine whether technology and screen media use were significant predictors of symptom severity category at baseline for all mental health symptoms. CLMs included age at baseline (in years) and sex (female and not female) as covariates, and total time of use as a main effect. The additional predictors of elevated use (True or False) of any screen media type (e.g., elevated use of video streaming) were added to the model if they improved model fit (see Supplementary Material 1.2.3, Table S1 for determination of all predictors). To address the potential of multicollinearity confounding model interpretation, the relationship between time using technology and elevated use of each screen media were assessed in pairwise correlations. Results from these analyses are shown in Supplementary Material Table S2. None of the pairwise correlations were strong (|R| ≥ 0.70), suggesting that multicollinearity is not a significant concern in our analyses.

For children with elevated screen media use at baseline—determined by elevated use of at least one type of screen media—we used longitudinal analyses to assess whether screen media use changed during care, and we also assessed associations between screen media use in mental health outcomes. Rates of decreases in elevated screen media use from baseline to last follow-up, as well as any assessment during care, were reported for all screen media types (a decrease in reported hours of use for any type constituted a “decrease in use”). Rates of non-elevated screen media use were also reported at last follow-up and during care. A linear mixed-effects model was used to determine whether elevated use of any screen media predicted mental health outcomes over care with the DMHI. Only children with mild to severe mental health symptoms (of that type) at baseline, as well as elevated screen media use, were included in this analysis. For each mental health symptom, the linear mixed-effects model of change in score (from baseline) included main effects of months in care (i.e., 30-day months from the date in care; continuous) and elevated screen media use (yes or no), as well as the interaction of months in care with elevated screen media use (yes or no). Age at baseline (in years) and sex (female and not female) were included as covariates, with a random effect of subject on the intercept (i.e., to account for the within-subject correlations of repeated measures). For all statistical analyses, the alpha-level was set at 0.05 (statistical significance), however *P*-values < .10 are also addressed to further characterize the data. Data were analyzed using R version 4.4.1 (46).

## 3 Results

### 3.1 Technology and screen media use at baseline

**Figure 1:**
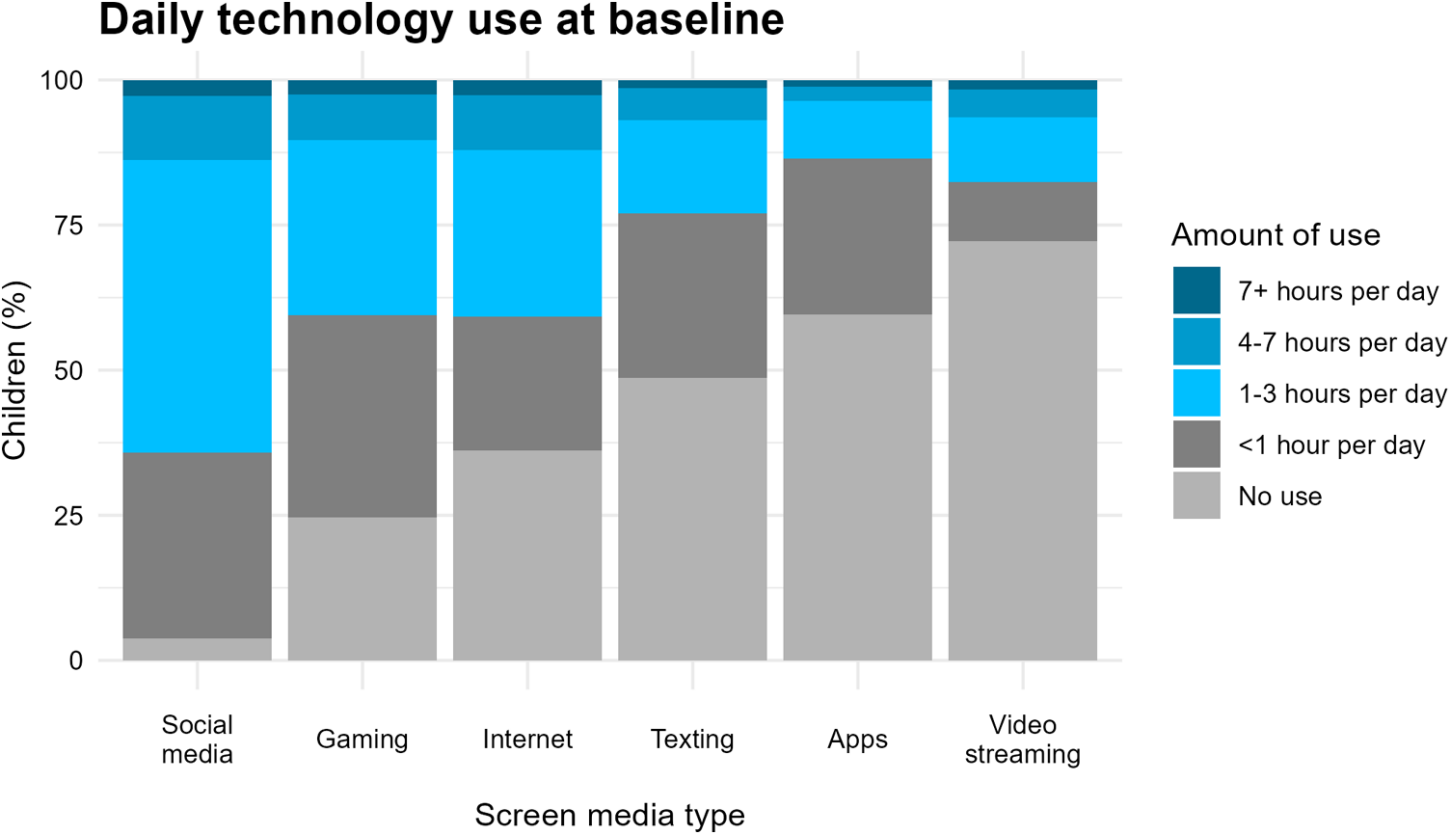
Amount of use per day for each type of screen media reported for children at baseline.

Children (*n* = 2,835) were 9.3 ± 1.9 years at baseline, and 46.5% (*n* = 1,319) were female. Over half (51.5%; *n* = 1,459) were White, 23.3% (*n* = 660) identified as other, and 9.5% (*n* = 270) were multi-racial. Children used technology and screen media for a median of three hours (2–5) per day at baseline. Only 2.0% (*n* = 56) of caregivers reported that their child did not use any technology (hours per day = 0). Problematic use of technology was reported by caregivers in 58.3% (*n* = 1,1654) of children. The rates of use of each screen media type are shown in Figure 1, and rates of any use and elevated use (4 + hours per day) are shown in Table 1. Nearly all children (96.2%; *n* = 2,727) used video streaming, with elevated use identified in 13.8% (*n* = 391), and approximately three in four (75.3%; *n* = 2,136) using gaming screen media (elevated use in 10.3%; *n* = 293). Children used a median of 4 (IQR: 2–5) different types of screen media, and elevated use of at least one type of screen media was identified in 23.2% (*n* = 657) of children.

**Table 1:** Rates of any use and elevated use of technology and screen media at baseline, reported by type of screen media.

Screen media type	% children with any use (*n* of 2,835)	% children with elevated use (*n* of 2,835)
Video streaming	96.2% (*n* = 2,727)	13.8% (*n* = 391)
Gaming	75.3% (*n* = 2,136)	10.3% (*n* = 293)
Apps	63.8% (*n* = 1,809)	12.0% (*n* = 341)
Internet	51.3% (*n* = 1,455)	6.9% (*n* = 197)
Texting	40.4% (*n* = 1,146)	3.6% (*n* = 102)
Social media	27.8% (*n* = 787)	6.4% (*n* = 182)

### 3.2 Technology and screen media use as a predictor of mental health

Table 2 shows the comprehensive results for technology and screen media use as a predictor of symptoms of mental health symptoms at baseline. For depression, greater time using technology and screen media was associated more severe symptoms (0.05 ± 0.02; z = 2.19, *P* = .022), as was elevated use of video streaming (0.31 ± 0.14; z = 2.23, *P* = .026), the internet (0.42 ± 0.18; z = 2.33, *P* = .020), and texting (0.48 ± 0.22; z = 2.17, *P* = .030). For anxiety, greater time using technology was associated with more severe symptoms (0.04 ± 0.01; z = 2.36, *P* = .019). For inattention, elevated video streaming (0.29 ± 0.13; z = 2.34, *P* = .020) and elevated internet use were associated with more severe symptoms (0.34 ± 0.17; z = 2.02, *P* = .043). For hyperactivity, elevated internet use was associated with more severe symptoms (0.41 ± 0.18; z = 2.34, *P* = .020), and the main effect of gaming approached statistical significance (0.26 ± 0.15; z = 1.71, *P* = .088). For opposition, there was a statistical trend towards greater time using technology predicting more severe symptoms (0.03 ± 0.02; z = 1.72, *P* = .086). Elevated use of gaming (0.30 ± 0.13; z = 2.32, *P* = .020) also predicted more severe oppositional symptoms, and the main effect of internet use approached statistical significance (0.28 ± 0.16; z = 1.79, *P* = .073). Regarding covariates, female sex predicted more severe depression (z = 2.94, *P* = .003) and anxiety (z = 5.80, *P* < .001), and less severe inattention (z = −7.15, *P* < .001), hyperactivity (z = −6.40, *P* < .001), and opposition (z = −4.77, *P* < .001). Older age predicted more severe depressive symptoms (z = 7.58, *P* < .001), and younger age predicted more severe symptoms of hyperactivity (z = −8.73, *P* < .001) and opposition (z = −5.04, *P* < .001). Other predictors were not statistically significant (*P* ≥ .10).

**Table 2:** Statistical results from the analyses of technology and screen media use and elevated use of each type of screen media as predictors of mental health symptoms at baseline.

Model term	Depression	Anxiety	Inattention	Hyperactivity	Opposition
	Z-statistic (*P*-value)	Z-statistic (*P*-value)	Z-statistic (*P*-value)	Z-statistic (*P*-value)	Z-statistic (*P*-value)
Main effects					
Time using technology	**2.92 (*P*** **=** **.022)**	**2.36 (*P*** **=** **.019)**	0.95 (*P* = .34)	0.89 (*P* = .37)	*1.72 (P* *=* *.086)*
Video streaming	**2.23 (*P*** **=** **.026)**	NA	**2.34 (*P*** **=** **.020)**	1.48 (*P* = .14)	NA
Gaming	NA	NA	1.55 (*P* = .12)	*1.71 (P* *=* *.088)*	**2.32 (*P*** **=** **.020)**
Apps	0.77 (*P* = .44)	NA	*0.19 (P* *=* *.85)*	0.26 (*P* = .15)	NA
Internet	**0.23 (*P*** **=** **.019)**	NA	**2.02 (*P*** **=** **.043)**	**2.34 (*P*** **=** **.020)**	*1.79 (P* *=* *.073)*
Texting	**2.17 (*P*** **=** **.030)**	NA	NA	NA	1.54 (*P* = .12)
Social media	NA	NA	NA	NA	NA
Covariates					
Age at baseline	**7.58 (*P*** **<** **.001)**	*1.92 (P* *=* *.055)*	0.64 (*P* = .52)	**−8.73 (*P*** **<** **.001)**	**−5.04 (*P*** **<** **.001)**
Female sex	**2.94 (*P*** **=** **.003)**	**5.80 (*P*** **<** **.001)**	**−7.15 (*P*** **<** **.001)**	**−6.40 (*P*** **<** **.001)**	**−4.77 (*P*** **<** **.001)**

Main effects that were not included in each respective model are indicated by “NA”. *P*-values <.05 are bolded, and *P*-values <.10 are italicized.

Table 3 reports all results for the type of screen media as a moderator of the relationship between time using technology and symptom severity. In terms of screen media type as a moderator for associations between time using technology and depressive symptoms, there was a statistical trend for video streaming (z = −1.69, *P* = .090) and internet use (z = 1.81, *P* = .070). These results suggest a marginally weaker association between time using technology and depression for those with elevated video streaming and a marginally stronger association between technology use and depression for those with elevated internet use. For inattention and hyperactivity, all interactions were not significant. For opposition, the interactions between internet use (z = 1.84, *P* = .066) and texting (z = −1.91, *P* = .056) with time using technology trended towards significance. These results suggest a marginally stronger association between time using technology and opposition for those with elevated internet use and a marginally weaker association between technology use and opposition for those with elevated texting.

**Table 3:** Statistical results from the analyses of elevated use of each type of screen media as a moderator of the relationship between time using technology and symptom severity at baseline.

Model term	Depression	Inattention	Hyperactivity	Opposition
	Z-statistic (*P*-value)	Z-statistic (*P*-value)	Z-statistic (*P*-value)	Z-statistic (*P*-value)
Main effects				
Time using technology	**2.91 (*P*** **=** **.004)**	*1.83 (P* *=* *.068)*	0.46 (*P* = .65)	0.91 (*P* = .37)
Video streaming	**2.50 (*P*** **=** **.013)**	**2.51 (*P*** **=** **.012)**	*0.52 (P* *=* *.60)*	NA
Gaming	NA	1.19 (*P* = .23)	1.00 (*P* = .32)	0.18 (*P* = .86)
Apps	*1.68 (P* *=* *.094)*	0.14 (*P* = .89)	NA	NA
Internet	−0.66 (*P* = .51)	0.28 (*P* = .78)	0.60 (*P* = .55)	−0.92 (*P* = .36)
Texting	1.34 (*P* = .18)	NA	NA	**2.32 (*P*** **=** **.021)**
Social media	NA	NA	NA	NA
Interactions *(time using technology X screen media type)*				
Video streaming	*−1.69 (P* *=* *.090)*	−0.41 (*P* = .68)	0.24 (*P* = .81)	NA
Gaming	NA	NA	−0.29 (*P* = .77)	0.95 (*P* = .345)
Apps	−1.54 (*P* = 0.13)	1.47 (*P* = .14)	NA	NA
Internet	*1.81 (P* *=* *.070)*	−0.57 (*P* = .57)	0.49 (*P* = .63)	*1.84 (P* *=* *.066)*
Texting	−0.37 (*P* = .71)	NA	NA	*−1.91 (P* *=* *.056)*
Social media	NA	NA	NA	NA
Covariates				
Age at baseline	**7.24 (*P*** **<** **.001)**	0.35 (*P* = .73)	**−8.55 (*P*** **<** **.001)**	**−4.92 (*P*** **<** **.001)**
Female sex	**2.98 (*P*** **=** **.003)**	**−7.09 (*P*** **<** **.001)**	**−6.42 (*P*** **<** **.001)**	**−4.76 (*P*** **<** **.001)**

Main effects and interactions that were not included in each respective model are indicated by “NA”. *P*-values <.05 are bolded, and *P*-values <.10 are italicized.

### 3.3 Longitudinal analysis of change in screen media use and mental health symptoms during mental health care

Children with elevated use of any type of screen media (*n* = 418) took their last technology and screen media use survey a median of 3.02 months (IQR: 1.40–5.03) after beginning care with the DMHI. At this time, 96.7% (*n* = 404 of 418) had participated in coaching, and 42.6% (*n* = 178) had participated in coaching. At any point during care with the DMHI, 93.1% (*n* = 389) had a decrease in screen media use and 69.1% (*n* = 289) did not have elevated use of any type of technology. At their last assessment, 84.2% (*n* = 352) had a decrease in use and 55.3% (*n* = 231) did not have elevated use of any type of technology. Rates of decreases in use and rates of non-elevated use during care differed based on the type of screen media considered (see Supplementary Material Table S3).

Table 4 includes comprehensive results from the analyses of technology and screen media use as a moderator of symptom improvement during care with the DMHI. Mental health symptom severity decreased over months in care for all symptoms, though this main effect did not reach statistical significance for inattention symptoms (z = −1.73, *P* = .085; all other symptoms *P* < .05). Elevated screen media use predicted more severe symptoms for depression (z = 2.79, *P* = .006), anxiety (z = 3.82, *P* < .001), and inattention (z = 2.33, *P* = .020). Notably, the interaction between months in care and screen media use approached significance for anxiety (z = −1.87, *P* = .062) and inattention (z = −1.90, *P* = .058), suggesting that children with elevated screen media use had larger improvements in anxiety and inattention symptoms than children with non-elevated screen media use. Age at baseline was significant for all mental health symptoms (all *P* < .05), and female sex predicted more severe symptoms of inattention (z = 2.67, *P* = .008).

**Table 4:** Statistical results from the analyses of technology and screen media use as a moderator of mental health outcomes during care with the DMHI.

Model term	Depression (*n* = 163)	Anxiety (*n* = 209)	Inattention (*n* = 232)	Hyperactivity (*n* = 138)	Opposition (*n* = 239)
	Z-statistic (*P*-value)	Z-statistic (*P*-value)	Z-statistic (*P*-value)	Z-statistic (*P*-value)	Z-statistic (*P*-value)
Main effects					
Months in care	**−3.34 (*P*** **<** **.001)**	**−4.35 (*P*** **<** **.001)**	*−1.73 (P* *=* *.085)*	**−2.03 (*P*** **=** **.043)**	**−4.13 (*P*** **<** **.001)**
Elevated screen media use	**2.79 (*P*** **=** **.006)**	**3.82 (*P*** **<** **.001)**	**2.33 (*P*** **=** **.020)**	1.26 (*P* = .21)	1.24 (*P* = .22)
Interaction					
Months in care X Elevated screen media use	−1.10 (*P* = .27)	*−1.87 (P* *=* *.062)*	*−1.90 (P* *=* *.058)*	−0.81 (*P* = .42)	−0.05 (*P* = .96)
Covariates					
Age at baseline	**−2.28 (*P*** **=** **.024)**	**−2.35 (*P*** **=** **.020)**	**−4.55 (*P*** **<** **.001)**	**−2.01 (*P*** **=** **.047)**	**−2.10 (*P*** **=** **.037)**
Female sex	0.62 (*P* = .54)	1.27 (*P* = .21)	**2.67 (*P*** **=** **.008)**	0.71 (*P* = .48)	1.32 (*P* = .19)

*P*-values <.05 are bolded, and *P*-values <.10 are italicized.

## 4 Discussion

The purpose of this study was to understand technology and screen media use in children seeking treatment from a DMHI, identify associations between these behaviors and mental health symptom severity at baseline, and explore how elevated use of screen media is associated with treatment outcomes longitudinally over the course of the DMHI. Almost all of the children (98.0%) used screen media of any type, more than half (58.3%) exhibited problematic use of technology, and elevated use of screen media was identified in approximately one in four (23.2%). At baseline, greater time using technology and screen media predicted more severe symptoms of depression and anxiety, though the specific types of screen media use linked to symptom severity differed based on mental health symptom type. Screen media use decreased for most (93.1%) children with elevated use at baseline, and 69.1% reported non-elevated use during care. While symptoms of depression, anxiety and inattention were more severe during care for those with elevated technology use, we found preliminary evidence of marginally greater improvements in inattention and anxiety symptoms when caregivers reported elevated technology use.

Consistent with existing evidence that children spend a significant amount of time on screen media (1, 2, 47), almost all children in the present study had some form of screen media use and a majority exhibited problematic use of technology. Screen media use was exacerbated by the COVID-19 pandemic, with both overall screen time and the proportion of children spending more than two hours per day on screens increasing during the pandemic (48–50). Screen time has remained elevated in recent years (51). Our findings align with current trends in children's screen media use, but more research is needed to understand its impact on their development and well-being.

Most children in the present study used multiple types of technology, with most using video streaming (96.2%) and gaming (75.3%). Fewer than half of the participants in this study used social forms of screen media, with 40.4% texting and 27.8% on social media. This is consistent with a recent systematic review that found that children ages 6–14 years mainly use screens for entertainment purposes rather than for social interaction (49). This disparity highlights a potential area for intervention, as the predominance of passive over active screen use may contribute to mental health issues (31, 32). However, not all entertainment-based screen media use is passive; for example, gaming can be both interactive and socially engaging. Recognizing these distinctions and promoting a balance between passive and active screen use may help mitigate potential adverse effects on children's emotional and social development.

Greater time spent using technology and screen media was associated with more severe depressive and anxiety symptoms at baseline, though associations with time using specific types of screen media differed between the two outcomes. Our findings align with literature showing that overall screen media time is linked to worse mental health outcomes in youth (27). For depressive symptoms, overall time and specific types of screen activity, including video streaming, internet browsing, and texting, were associated with symptom severity. This aligns with broader research indicating that certain types of screen media may exacerbate depressive symptoms more than others (33, 34), with newer technologies like mobile phones and internet use showing stronger links to depressive symptoms than older forms such as television watching (33). Targeted interventions addressing high-risk screen activities may be more effective than reducing overall screen time to mitigate depressive symptoms in children. In contrast, for anxiety, while greater time spent using screen media was associated with symptom severity, the relationship was not specific to specific types of screen media. Although this finding aligns with prior evidence demonstrating that screen media use in children ages 9–10 impacted anxiety symptoms two years later (12), it underscores the need for further research to clarify how patterns of technology and screen media use may influence anxiety symptoms. Notably, not all screen media engagement is inherently harmful. Some forms of digital interaction may provide cognitive, emotional, or social benefits. Research suggests that the impact of screen media on mental health depends on factors such as content, purpose, and context of use, rather than screen time alone (17, 18, 52, 53). Indeed, given the heterogeneity of findings regarding associations between screen media use and pediatric outcomes, future research should focus on disentangling these complexities to better inform interventions that support adaptive, rather than restrictive, technology use.

Although we found a statistical trend linking overall screen media use to more severe oppositional symptoms at baseline, no such association emerged for inattention or hyperactivity. Our findings align with studies associating higher screen time with ODD in adolescents and new-onset conduct problems in children aged 9–11 years (14, 54). Our lack of findings regarding inattention and hyperactivity may reflect complexities in how overall screen media use relates to these symptoms, as ADHD symptoms have been shown to be bidirectionally and longitudinally linked to screen media use in children and adolescents (15). Notably, elevated use of video streaming and internet activities predicted greater inattention, while elevated internet use was linked to hyperactivity, and gaming was associated with oppositional symptoms. Children with ADHD and problematic digital media use have been shown to have more severe inattention, oppositional behaviors, and emotional difficulties, particularly with excessive video game and social media use, emphasizing the need to address specific types of screen media in managing ADHD-related symptoms (55). Digital media use may impair attentional performance, particularly when media distractions are perceived as more important than the primary task or involve sensory overlap, though the long-term effects of media multitasking on sustained attention remain unclear (56). These findings underscore the importance of examining specific screen media activities to better understand their unique contributions to behavioral symptoms and guide targeted interventions.

During care, elevated technology and screen media use were associated with more severe symptoms of depression, anxiety, and inattention among individuals with elevated technology use, aligning with prior studies linking higher technology engagement to greater mental health challenges (14, 15, 22–27). This is particularly relevant for today's youth, often referred to as “digital natives” due to their lifelong familiarity with digital technologies (57). While the interventions did not specifically target screen time reduction, it is possible that improvements in mental health symptoms contributed to more regulated screen media use over time. For instance, reductions in anxiety and inattention may have allowed for better self-regulation, decreasing excessive or dysregulated screen engagement. Additionally, therapeutic components of the DMHI—such as CBT, DBT, and parent management strategies—may have indirectly influenced screen media habits by improving emotion regulation and caregiver involvement in setting boundaries around technology use. Research suggests that children's media use and self-regulation are closely linked, not only through direct effects but also through parent-child interactions, where caregiver involvement and modeling of screen habits play a key role in shaping children's ability to regulate their own technology use (58). Understanding these dynamics is important for future research and intervention development, as fostering self-regulation skills and promoting healthy parent-child interactions may play a role in supporting more balanced and intentional screen media use among children.

We found preliminary evidence of marginally greater improvements in inattention and anxiety symptoms among those whose caregivers reported elevated technology use during care with the DMHI. It is possible that being digital natives makes these individuals more inclined to engage with the DMHI, leading to greater interaction with therapeutic content delivered through screen media. The type of therapeutic content within the DMHI may have also contributed to these improvements. For example, CBT and DBT involve structured skill-building exercises that could reinforce focus and self-regulation, while mindfulness-based interventions may have helped manage anxiety by promoting intentional digital engagement. Additionally, parent management strategies may have influenced screen media habits at home, shaping how children engaged with both recreational and therapeutic digital content. While we asked about time spent using specific types of screen media use, such as internet use, we did not assess what children were specifically doing during these activities. This lack of detail limits our ability to determine whether certain activities—such as engaging with therapeutic content from the DMHI, social interactions, or leisure pursuits—may have influenced symptom trajectories. With consideration to the debated literature showing that use of screen media is not inherently harmful (17, 18, 52, 53), future research should investigate the specific activities associated with each type of screen media, including the proportion of time spent engaging with DMHI-related content, to better understand the nuanced role of technology use in symptom improvement and DMHI outcomes.

These findings suggest that future DMHIs should consider how children's existing technology and screen media habits interact with treatment engagement and outcomes. Given that children with elevated screen media use showed both greater mental health symptom severity and marginal improvements in inattention and anxiety, digital interventions may need to differentiate between beneficial and problematic forms of screen use. Designing DMHIs that integrate structured, intentional digital engagement— while promoting healthy screen habits— could enhance effectiveness, particularly for children who are already highly immersed in technology. However, more research is needed to corroborate this.

### 4.1 Strengths and limitations

The findings presented here should be considered within the strengths of this study. First, this study contributes to the growing body of evidence on the relationship between technology and screen media use and mental health symptoms in the context of a collaborative care DMHI, offering valuable insights into their broader impact. The present study focuses on a treatment-seeking population of children within the context of an intervention that is, in itself, delivered via technology, significantly adding to our understanding of the nuances in associations between screen media and health. Second, this study differentiates between various types of screen media, such as gaming, social media, and video streaming, rather than solely exploring total screen time. This provides a more nuanced understanding of how different forms of screen media may uniquely impact children's mental health. Third, the study benefits from the use of comprehensive depressive and anxiety symptom assessments, with validated tools used to quantify symptom severity. These measures, as well as screen media use, were measured frequently over the course of treatment, allowing for a more detailed understanding of symptom progression and technology habits. Fifth, the study has a large sample size at baseline, enhancing the generalizability and robustness of the findings. Lastly, this is the first study, to our knowledge, to explore how children's screen media habits interact with their engagement in a DMHI, providing valuable insights into how these habits may impact treatment outcomes. This insight provides an opportunity to refine DMHIs by considering how children's broader screen media habits interact with treatment, ultimately paving the way for more tailored and effective interventions.

Our findings are limited by several factors. In terms of generalizability, the sample consists of children with access to a DMHI, which may not reflect the experiences or outcomes of those with more restricted access to technology. This study also did not account for all potential predictors of child mental health outcomes, including caregiver involvement, socioeconomic status, extracurricular engagement, and physical health outcomes, which further limits generalizability. However, it is important to note that caregivers are required to be involved in care for children who receive treatment at Bend. Despite this, without accounting for additional inter-individual factors, it is unclear whether the observed effects may differ by family factors, non-technology engagement, or overall health. Future research should consider these variables when evaluating treatment outcomes. Considerations should also be made for the measures we leveraged for this study. Although there is precedent for caregiver-reported outcomes (59–61), caregivers completed all assessments on both child mental health symptoms and screen media use, which may not capture internalizing symptoms or specific media types as accurately as child self-reports (6, 61). Additionally, caregiver-reported social media use and mental health symptoms may introduce bias and inaccuracies due to recall errors, social desirability effects, or subjective interpretations of screen use. The reliance on caregiver reports in this study highlights a broader challenge with pediatric research, as findings on children's digital experiences often reflect an adult-centered perspective, which may overlook children's agency and firsthand experiences (58). It should be noted that, while the caregiver is encouraged to complete assessments with their child present to weigh-in on responses, the exclusion of the child's unbiased perspective is a major concern for research (such as this), which attempts to describe child behavior from a proxy-report. Future studies should consider incorporating objective measures (e.g., device monitoring), as well as both child self-report and caregiver-report assessments to determine if these results are consistent across various measures.

In terms of study design, this study is retrospective, so any conclusions regarding the role of screen media use in mental health treatment cannot be considered causal. That is, we cannot definitively assert that our findings are a direct result of engagement with the DMHI, as would be possible with a randomized control trial. Further, beyond concerns about caregiver-reported technology outcomes, the technology and screen media screener and survey reported here are investigator developed. Although we based the development of the questions based on prior research (40, 41), our measure is limited in its reliability and validity. Using objective measures and validated assessments in future studies is recommended. Additionally, the survey likely lacks specificity, as it did not differentiate between screen use for educational vs. recreational purposes, and there may have been some overlap in some of the categories (e.g., watching videos on social media platforms). Similarly, ‘Internet use’ was not clearly distinguished from video streaming and other screen activities, which may have led to some variability in caregiver interpretation. However, since the same caregiver completed the assessments at each time point, their reporting was likely consistent over time. To ensure results were not skewed by confusion over categorization of screen media use, the authors did not use survey responses to estimate total time spent using screen media, as others have (40). Future research should account for these nuances in screen media use to parse apart associations between more specific patterns of use. While the sample size was large at baseline, it was smaller for models assessing changes in symptom severity, reducing statistical power. We could not compare changes in screen media use across different forms of mental health treatment (e.g., face-to-face therapy, other DMHIs, no intervention), limiting conclusions about its effects within various therapeutic contexts. Future studies should include multiple treatment modalities for a more comprehensive analysis. Finally, all authors were employed by or contracted with Bend Health Inc., which delivered the treatment used in this retrospective study. However, the authors’ employment status and compensation (e.g., salary) were not, and are not, dependent on the findings or publication of their research.

## 5 Conclusion

This study highlights the complex relationships between technology and screen media use and mental health symptoms in children receiving care through a collaborative care DMHI. Elevated screen media use was linked to more severe depressive, anxiety, and ADHD-related symptoms, with specific types, such as video streaming, internet use, and gaming, showing unique associations with symptom severity. Notably, technology and screen media use decreased for most children during care, and elevated technology use was associated with marginally greater improvements in inattention and anxiety symptoms. These findings underscore the need for DMHIs to account for children's screen media habits, leveraging their digital engagement to enhance treatment outcomes while addressing the potential adverse effects of specific types of technology use.

## Data Availability

The data analyzed in this study is subject to the following licenses/restrictions: the data analyzed for this paper (retrospectively) include electronic health records of children. Therefore, the dataset cannot be made publicly available. The authors will take any reasonable requests for deidentified data under consideration. Requests to access these datasets should be directed to darian.lawrence@bendhealth.com.
