# Supplementary material for: The role of technology and screen media use in treatment outcomes of children participating in a digital mental health intervention: a retrospective analysis of Bend Health
Source: Front Digit Health. 2025 May 2;7:1556468. doi: 10.3389/fdgth.2025.1556468 (PMC12081425; doi:10.3389/fdgth.2025.1556468)
Supplement: Supplementary file 1 [file Supplementaryfile1.pdf]

Supplementary Material

1 Supplementary Methods

1.1 Measures

At enrolment, the response options to the demographic question about child race/ethnicity were: "White", "Black or African American", "American Indian or Alaska Native", "Chinese", "Vietnamese", "Native Hawaiian", "Filipino", "Korean", "Japanese", "Chamorro", "Other Asian", "Other Pacific Islander", "Some other race or multi-racial", "Mexican, Mexican American, or Chicano", "Puerto Rican", "Cuban", "Another Hispanic, Latino, or Spanish origin." Multi-select is allowed on this question. For reporting purposes, these responses were grouped into larger descriptive categories. A reported race/ethnicity of "Asian" included the responses: "Chinese", "Vietnamese", "Filipino", "Korean", "Japanese", "Chamorro", "Other Asian", or "Other Pacific Islander." A reported race/ethnicity of "Hispanic or Latino" included the responses: "Mexican, Mexican Am., Chicano", "Puerto Rican", "Cuban", "Another Hispanic, Latino, or Spanish origin." Members with a reported race/ethnicity of "Other" and those that selected multiple race/ethnicity options, were categorized as "Other or multi-racial". Otherwise, the responses were not re-categorized for reporting. As of January 23rd, 2024, caregivers are automatically given the comprehensive survey if they screened-in to the survey in any previous assessments (automatic screen-in). Between January 23rd, 2024, and August 9th, 2024, the screener questions were not repeated at each follow-up if the child had already screened in.

1.1.1 Technology and screen media survey

The second screener question asks: "On a typical day, how many hours per day does your child spend using technology" (whole-number numerical response 0-24). Caregivers complete the remaining questions of the survey if they indicate problematic use of technology ("Yes" to the first question) or greater than 0 hours of technology use per day (screen-in). These remaining questions query about daily use (within the past month) of texting, social media, other smart-phone apps, the internet, watching or streaming movies/videos/TV, and gaming, for example: "How much time does your child spend texting?" The response options are as follows: "My child does not use this type of technology" (score=0), "1 hour or less a day" (score=1), "More than 1 hour but less than 4 hours" (score=2), "More than 4 hours but less than 7 hours" (score=3), "More than "7 hours" (score=4). For caregivers of children who screen-in and complete the entire survey, the final six questions about types of screen media are repeated at each follow-up survey thereafter. If caregivers of children are not screened-in, they first respond to the screening questions after each follow-up survey, and then may answer the final six questions (i.e., if they screen-in).

1.1.2 Mental health symptom severity

To screen for depressive symptoms, caregivers respond to the two screening questions inquiring whether their child "had less fun doing things than they used to" and "seemed sad or depressed for several hours" in the past two weeks. To screen for anxiety symptoms, caregivers respond to the three screening questions inquiring whether the child "said they felt nervous, anxious, or scared," has "not been able to stop worrying", and "said they couldn't do things they wanted to or should have done, because it made them feel nervous" in the past two weeks. The PROMIS Emotional Distress

assessments for depression and anxiety inquire about the frequency of a child displaying common symptoms of depression or anxiety, respectively, in the past seven days. The depression assessment is 11-items, including questions about feelings of sadness and loneliness, as well as social isolation.1,2 The anxiety assessment is 10-items, including questions about feelings of nervousness and worry.1,2 Caregivers respond to the inattention/hyperactivity and opposition screener questions based on their child's behavior in the past two weeks. The SNAP-IV assessment is 26-items, including subsets to measure inattention (items 1-9), hyperactivity (items 10-18), and opposition (items 19-26)3. Before April 1st, 2024, a screen-in response (1 or greater) on the inattention/hyperactivity screener question prompted questions 1-18 of the SNAP-IV, and a screen-in response (1 or greater) on the opposition screener question prompted questions 19 to 26 of the SNAP-IV. Starting April 1st, 2024, both screener questions screened for the entire SNAP-IV questionnaire.

1.2 Statistical analysis

1.2.1 Participant inclusion

For technology and screen media use at baseline, all eligible children (N=2,861) were included in analyses. For technology and screen media use as a predictor of mental health, all children with a complete screener or mental health assessment taken the say day as their baseline technology and screen media use survey were included in the analysis (Depression: n=2,720; Anxiety: n=2,749; Inattention: n=2,806; Hyperactivity: 2,806; Opposition: 2,630). For change in screen media use and mental health symptoms during mental health care, children with elevated use of at least one type of screen media at baseline and at least one follow-up technology and screen media survey (after care start) were included in the analysis of change in screen media use (n=418). For the analysis of change in mental health symptoms during mental health care, a subset of these participants were analyzed, including only those with mild to severe symptoms at baseline as well as at least one follow-up assessment after the start of care (Depression: n=163; Anxiety: n=209; Inattention: n=232; Hyperactivity: 138; Opposition: 239).

1.2.2 Mental health score calculations

Depression and anxiety aggregate assessment scores were converted to T-scores per standardized procedures, with the following symptom severity categories 1,2: none to slight (screened-out assessments and T-scores < 55), mild (T-scores 55.0 to 59.9), moderate (T-scores 60.0 to 69.9), and severe (T-scores ≥ 70). Inattention, hyperactivity, and opposition scores were aggregated, and then assigned symptom severity categories. For inattention and hyperactivity, severities were: none to slight (screened-out assessments and scores < 13), mild (scores 13 to 17), moderate (scores 18 to 22) and severe (scores > 22). For opposition, severities were: none to slight (screened-out assessments and scores < 8), mild (scores 8 to 13), moderate (scores 14 to 18), and severe (scores > 18).

1.2.3 Models

The basic cumulative link model (CLM) for analysis of technology and screen media use as a predictor of mental health symptom severity (at baseline; None to slight, mild, moderate, severe) included time of use as a predictor, as well as age at baseline (in years) and sex (female and not female) as covariates for all mental health symptom types. The predictor of elevated use (True or False) of any screen media type was added one at a time to the basic model as a potential predictor, and then the fit of each of these potential models were compared to the basic model using likelihood

ratio test (LRT). Where a potential model had better fit than the basic model (i.e., a statistically significant LRT), the added predictor was retained in the final model. The results from each LRT are shown in Table S1. Z-values and corresponding P-values were reported for each predictor to determine whether each factor significantly associated with mental health symptom severity. To check for the possibility of multicollinearity confounding these results, correlations between time using technology and elevated use of each screen media type, as well as correlations between screen media types, were computed; see Table S2. Indeed, all R2-values were < .60, thus the analyses used here were appropriate and likely not confounded by multicollinearity. Z-tests were reported for each main effect and covariate to report whether time using technology and elevated use of screen media predicted mental health symptoms. Each model was repeated with the interaction of elevated use of screen media (each predictor retained) with time using technology included in the model. All Z-tests were reported, with a focus on the interaction terms to determine whether type of screen media moderates the relationship between time using technology and symptom severity. For the linear mixed-effects model of change in mental health symptoms, change in score from baseline (or T-score), as appropriate, was analyzed over months in care (calculated as 30-day months from first event with the DMHI). Only assessments after the start of care with the DMHI were analyzed.

2 Tables

Table S1. LRT results comparing models with each additional predictor to a basic model, with only time of use and demographic covariates. Predictors (elevated use of each type of screen media) that significantly improved model fit (P<.05) were retained in the final model (bolded text).

**Predictor**	**Depression**<br>LR= , P=	**Anxiety**<br>LR= , P=	**Inattention**<br>LR= , P=	**Hyperactivity**<br>LR= , P=	**Opposition**<br>LR= , P=
**Video streaming**	**LR_1=11.86, P<.001**	LR_1=1.84, P=.18	**LR_1=11.64, P<.001**	**LR_1=6.58, P=.010**	LR_1=0.66, P=.42
**Gaming**	LR_1=0.30, P=.58	LR_1=0.00, P=.95	**LR_1=6.75, P=.009**	**LR_1=6.27, P=.012**	**LR_1=7.11, P=.008**
**Apps**	**LR_1=11.00, P<.001**	LR_1=0.14, P=.70	**LR_1=4.98, P=.026**	LR_1=0.63, P=.43	LR_1=0.70, P=.40
**Internet**	**LR_1=17.54, P<.001**	LR_1=0.95, P=.33	**LR_1=9.50, P=.002**	**LR_1=9.16, P=.002**	**LR_1=7.00, P=.008**
**Texting**	**LR_1=11.94, P<.001**	LR_1=0.49, P=.48	LR_1=0.57, P=.45	LR_1=2.14, P=.14	**LR_1=5.08, P=.024**
**Social media**	LR_1=3.17, P=.075	LR_1=1.62, P=.20	LR_1=0.22, P=.64	LR_1=0.27, P=.60	LR_1=0.01, P=.91

Supplementary Material

Table S2. Pairwise correlations between total time using technology and use of each screen media type (elevated and non-elevated) for children at baseline to assess for multicollinearity.

	**1.**	**2.**	**3.**	**4.**	**5.**	**6.**	**7.**
**1. Time using technology**	**-**						
**2. Elevated use of video streaming**	**0.51**	**-**					
**3. Elevated use of gaming**	**0.43**	**0.41**	**-**				
**4. Elevated use of apps**	**0.50**	**0.48**	**0.40**	**-**			
**5. Elevated use of internet**	**0.41**	**0.39**	**0.29**	**0.46**	**-**		
**6. Elevated use of texting**	**0.30**	**0.20**	**0.15**	**0.37**	**0.45**	**-**	
**7. Elevated use of social media**	**0.41**	**0.30**	**0.22**	**0.54**	**0.36**	**0.44**	**-**

Table S3. Rates of decrease in screen media use and non-elevated use during care and at the last assessment reported for children with elevated use at baseline.

		**All assessments during care**		**Last assessment during care**	
**Screen media type**	**Elevated use at baseline**	**Decrease in use**	**Non-elevated use**	**Decrease in use**	**Non-elevated use**
Video streaming	n=250	88.4% (n=221)	82.4% (n=206)	74.4% (n=186)	68.0% (n=160)
Gaming	n=196	84.2% (n=165)	77.6% (n=152)	70.4% (n=152)	62.2% (n=122)
Apps	n=207	85.5% (n=177)	83.1% (n=172)	73.4% (n=138)	69.1% (n=143)
Internet	n=120	84.2% (n=101)	81.7% (n=98)	72.5% (n=87)	69.2% (n=83)
Texting	n=51	86.3% (n=44)	80.4% (n=41)	76.5% (n=39)	72.5% (n=37)
Social media	n=113	84.1% (n=95)	79.6% (n=90)	70.8% (n=80)	63.7% (n=72)
